# *HOT* Graphene and *HOT* Graphene Nanotubes: New Low Dimensional Semimetals and Semiconductors

**DOI:** 10.1186/s11671-020-3279-1

**Published:** 2020-03-06

**Authors:** Lin-Han Xu, Shun-Qing Wu, Zhi-Quan Huang, Feng Zhang, Feng-Chuan Chuang, Zi-Zhong Zhu, Kai-Ming Ho

**Affiliations:** 1 Department of Physics, Collaborative Innovation Center for Optoelectronic Semiconductors and Efficient Devices, Jiujiang Research Institute, Xiamen University, Xiamen, China; 2 Department of Physics, National Sun Yat-Sen University, Kaohsiung, 804 Taiwan; 3 Department of Physics and Astronomy, Ames Laboratory of DOE, Iowa State University, Ames, IA 50011 USA; 4 International Center for Quantum Design of Functional Materials (ICQD), University of Science and Technology of China, Hefei, 230026 Anhui China

**Keywords:** *HOT* graphene, Nanotubes, Electronic structures, First-principles calculations

## Abstract

We report a new graphene allotrope named *HOT* graphene containing carbon hexagons, octagons, and tetragons. A corresponding series of nanotubes are also constructed by rolling up the *HOT* graphene sheet. Ab initio calculations are performed on geometric and electronic structures of the *HOT* graphene and the *HOT* graphene nanotubes. Dirac cone and high Fermi velocity are achieved in a non-hexagonal structure of *HOT* graphene, implying that the honeycomb structure is not an indispensable condition for Dirac fermions to exist. *HOT* graphene nanotubes show distinctive electronic structures depending on their topology. The (0,1) *n* (*n* ≥ 3) *HOT* graphene nanotubes reveal the characteristics of semimetals, while the other set of nanotubes (1,0) *n* shows continuously adjustable band gaps (0~ 0.51 eV) with tube size. A competition between the curvature effect and the zone-folding approximation determines the band gaps of the (1,0) *n* nanotubes. Novel conversion between semimetallicity and semiconductivity arises in ultra-small tubes (radius < 4 Å, i.e., *n* < 3).

## Introduction

Because of its bonding flexibility, carbon-based systems show an unlimited number of different structures with an equally large variety of physical properties. These physical properties are, in great part, the result of the dimensionality of these structures [1]. Graphene is a single two-dimensional layer of carbon atoms bound in a hexagonal lattice structure [2] revealing a number of unique properties, such as massless carriers, high Fermi velocity [3], and Dirac cones [4, 5], which are characteristic of two-dimensional Dirac fermions. The honeycomb lattice consisting of two equivalent carbon sublattices plays a crucial role in forming such intriguing properties [2]. Enyashin and Ivanovskii [6] constructed 12 artificial 2D carbon networks but found no structures other than the graphyne allotrope exhibit the graphene-like electronic behavior. It seems to imply that the Dirac-like fermions in sp^2^-bonded carbon systems are dependent on the honeycomb structure. In the lower dimension, the carbon nanotube is a honeycomb structure rolled into a hollow cylinder with nanometric diameter and μm length [7–10]. As there is an infinite number of ways of rolling a sheet into a cylinder, the large variety of possible helical geometries, defining the tube chirality, provides a family of nanotubes with different diameters and microscopic structures [11–13]. The electronic and transport properties are certainly among the most significant physical properties of carbon nanotubes, and crucially depend on the diameter and chirality [14–18]. Graphene nanotubes can be either semimetallic [14] or semiconducting [19–21], with a band gap varying from zero to a few tenths of an eV, depending on their diameter and chirality [10, 14, 16]. Furthermore, the band gap of semiconducting tubes can be shown to be simply related to the tube diameter. The semimetallic nanotubes also maintain the unique properties from graphene, such as massless carriers, high Fermi velocity [22], and Dirac cones [23]. Such remarkable results can be obtained from a variety of considerations, starting from the so-called band-folding approach, based on knowledge of the electronic properties of the graphene sheet, to the direct study of nanotubes using semiempirical tight-binding approaches [14, 16, 18, 23]. Comparing with more sophisticated ab initio calculations and available experimental results, finer considerations, such as curvature effects, **k**_***F***_ shifting [24, 25], σ-π hybridization [26] are introduced. Graphene and graphene-like materials [6] are considered a revolutionary material for future generation of high-speed electronic, radio frequency logic devices [27, 28], thermally and electrically conductive reinforced composites [29, 30], catalyst [31], sensors [32–35], transparent electrodes [27, 36], etc. basing on the unusual properties all above. Over the past few decades, carbon nanotubes have also shown great potential in logic circuits, gas storage, catalysis, and energy storage because of their extraordinary electronic, mechanical, and structural properties [37–39]. Hence, the creation of new carbon allotropes (including 2D and 1D) has been the focus of numerous theoretical and experimental explorations because of their fundamental scientific and technological importance [40]. However, completely clarifying the structures of these exciting carbon phases through current experimental technologies is usually unrealistic due to their limited quantity, as well as the mixture of other phases. Theoretical prediction is necessary and has yielded great success [31–35, 40–42].

In this study, we designed a new allotrope of graphene that has two-dimensional Dirac fermions without an exclusively hexagonal structure. The new allotrope was constructed with interlaced carbon hexagons, octagons, and tetragons, and was named *HOT* graphene. *HOT* graphene nanotubes were also constructed by rolling up *HOT* graphene sheet along with different directions. The electronic property, curvature effect, **k**_***F***_ shifting effect, etc. of *HOT* graphene and nanotubes were calculated using ab initio calculations based on density function theory (DFT).

## Method of Calculation

The present calculations on *HOT* graphene and *HOT* graphene nanotubes were performed by using a first-principles method based on the density-functional theory (DFT) with the generalized gradient approximation (GGA) in the form of Perdew-Burke-Ernzerh (PBE) exchange-correlation functional [43], as implemented in the Vienna Ab initio Simulation Package (VASP) [44, 45]. The wave functions were expanded in plane waves up to a cutoff of plane wave kinetic energy of 520 eV. The Brillouin zone (BZ) integrals were performed by using a Monkhorst-Pack [46] sampling scheme with a **k**-point mesh resolution of 2π × 0.03 Å^− 1^. The unit cell basis vectors (representing unit cell shape and size) and atomic coordinates were fully relaxed in each system until the forces on all the atoms were smaller than 0.01 eV/Å.

## Results and Discussion

### Geometric and Electronic Structures of *HOT* Graphene

**Fig. 1:**
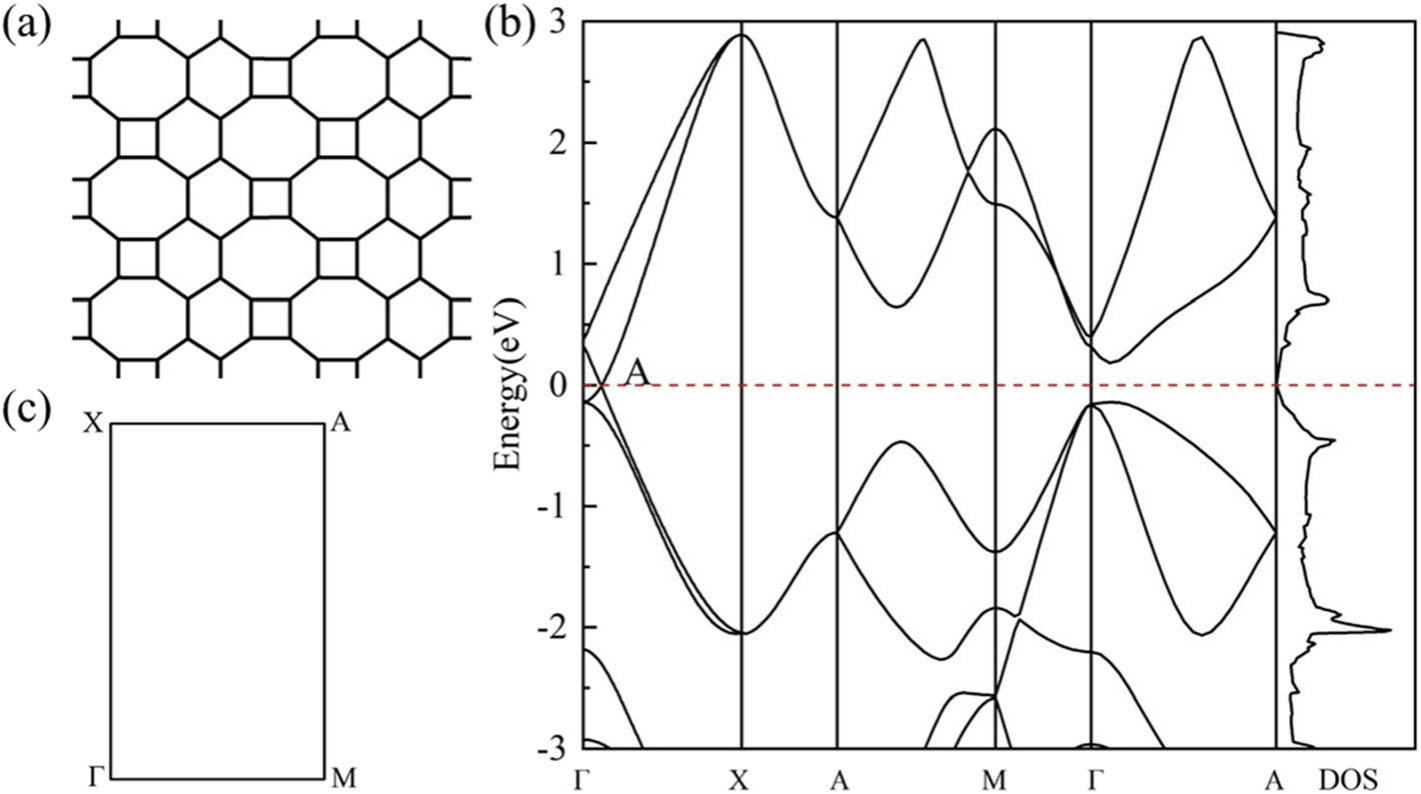
(**a**) Geometry of *HOT* graphene; (**b**) Band structures and DOS of the *HOT* graphene; (**c**) the corresponding BZ of *HOT* graphene

**Fig. 2:**
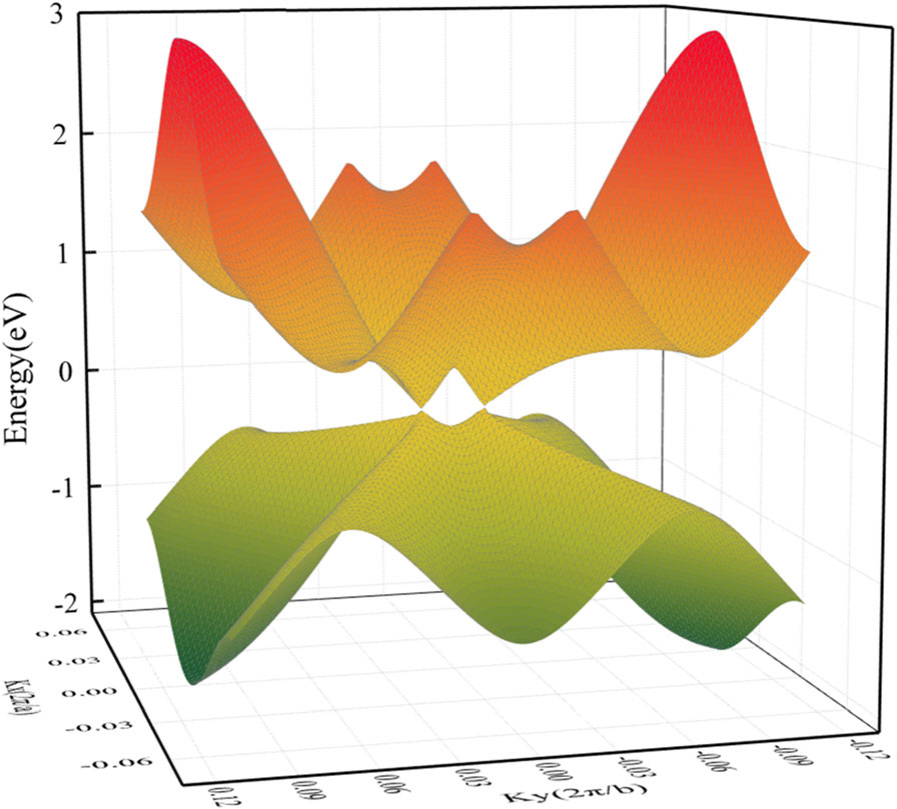
3D band structure of the *HOT* graphene

The geometric structure of *HOT* graphene (Fig. 1a) shows a more complicated bonding situation than graphene. The variety of carbon polygons in *HOT* graphene results in various carbon bonding characters. These polygons in *HOT* graphene share common edges with each other, and the bonds can be distinguished by the two polygons they belong to. Therefore, in our research, they are named as 6–8 bonds, 4–8 bonds, 4–6 bonds, 6–6 bonds, and 8–8 bonds. The 4–8 bonds and 6–8 bonds have two different bond lengths: 1.44 Å and 1.47 Å for 4–8 bonds; 1.41 Å and 1.48 Å for 6–8 bonds. The 4–6 bonds, 6–6 bonds, and 8–8 bonds have unique bond lengths of 1.44 Å, 1.46 Å, and 1.34 Å, relatively. Figure 1b shows the band structure and density of states (DOS) of *HOT* graphene with the corresponding BZ depicted in Fig. 1c. The crossing point of energy bands at the Fermi level indicates semimetallicity of *HOT* graphene, which is confirmed by the vanishing DOS at the Fermi level. The Dirac point is located at (0, 0.0585, 0) adjacent to Γ. The 3D band structure (Fig. 2) presents the band surfaces near the Fermi level, where one can see the Dirac cones formed by upper and lower conical surfaces meeting at two Dirac points exactly at the Fermi surface. The corresponding Fermi velocity (v_*F*_) of the Dirac fermions, evaluated from the gradient of the linear dispersions of the band structures, is 6.27 × 10^5^ m/s, which is a little lower than 8.1 × 10^5^ m/s [22] for graphene nanotube and 8.6 × 10^5^ m/s [47, 48] for graphene. The high v_*F*_ implies high mobility of carriers in the *HOT* graphene.

### Geometric and Electronic Structures of *HOT* Graphene Nanotubes

**Fig. 3:**
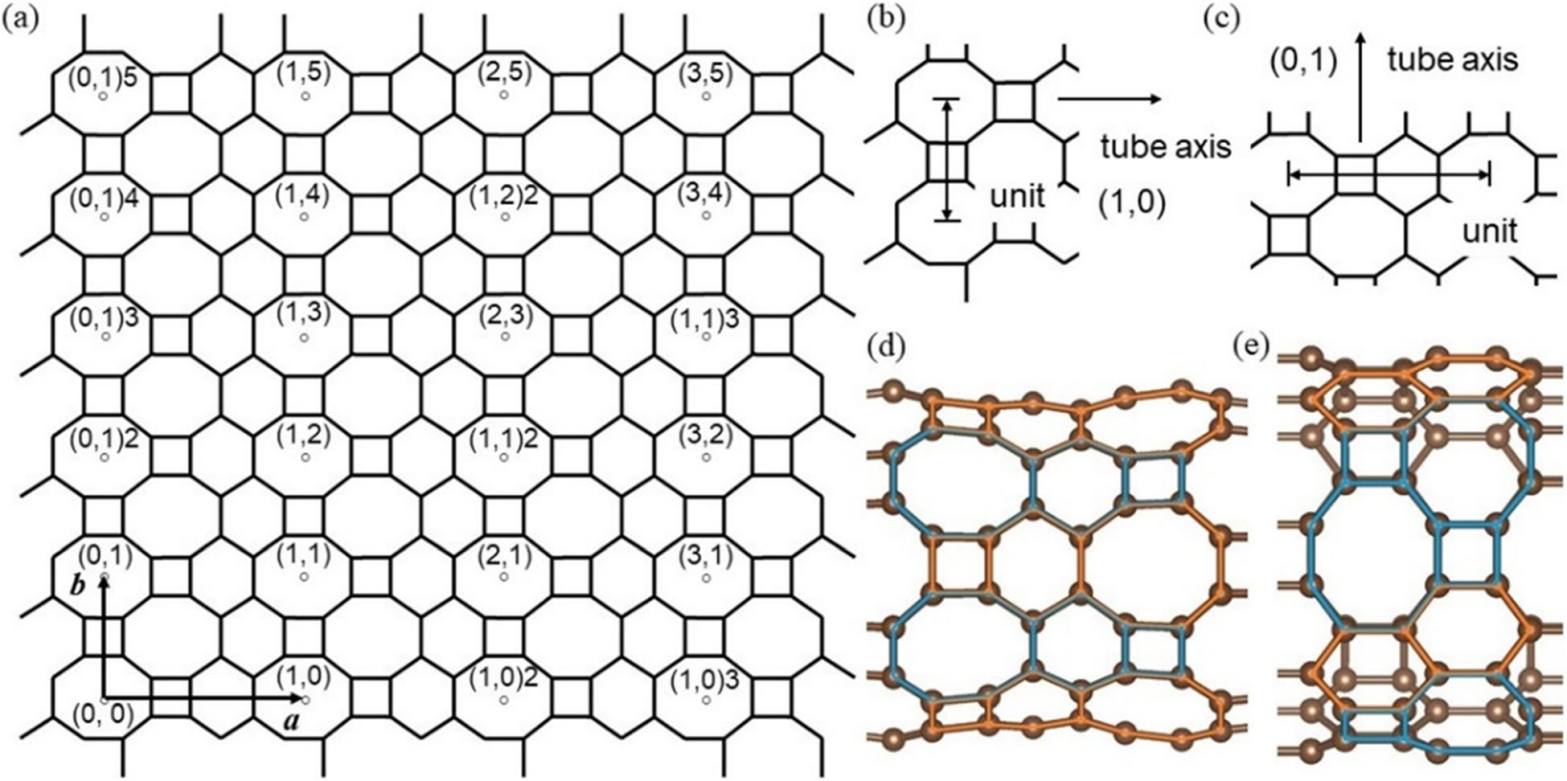
(**a**) Different tubes are denoted by symbol (*l,m*) *n*, with (*l,m*) stands for rolling direction based on unit vector ***a*** and ***b***, and *n* for the number of tube diameter units; (**b**) diameter unit on the (1,0) direction; (**c**) diameter unit on the (0,1) direction; (**d**) geometric structure of a (1,0)6 *HOT* graphene nanotube; (**e**) geometric structure of a (0,1)4 *HOT* graphene nanotube

The *HOT* graphene tubes are rolled up from the *HOT* graphene sheet in various directions symbolized by an index on the 2D *HOT* graphene lattice (Fig. 3a). This index is denoted as (*l,m*) *n* basing on two unit vectors ***a*** and ***b***. Symbol (*l,m*) [18, 20, 21] stands for different directions on the *HOT* graphene sheet, *n* stands for the number of perimeter units (Fig. 3b and c) used in rolling up the tubes. Under the space reversal symmetry of the *HOT* graphene unit cell, the nanotube symbol is confined to 0 ≤ *l* and 0 ≤ *m* to avoid a many-to-one correspondence. The number of possible geometric structures of the *HOT* graphene nanotubes is infinite since there are infinite choices for both diameters and rolling directions. Among such a large number of nanotubes, only two directions, (0,1, 1,0), are selected in our study. This is because the helical arrangement of atoms in other directions is barely periodic and possesses a nearly infinite length of unit cell along the tube axis. Such a large unit cell is unrealistic in our calculation. Different rolling directions between (1,0) *n* and (0,1) *n* tubes result in the differences in geometry and bonding situation. Two tubes, (1,0)6 and (0,1)4, are depicted in Fig. 3d and e to describe the geometric differences between the two rolling directions. The arrangement of polygons along (1,0) direction can be divided into two patterns: C_4_–C_6_–C_8_ (orange) and C_8_–C_6_–C_4_ (blue) which is exactly opposite to each other. These two opposite patterns alternate along the circumferential direction of the tube. In direction (0,1), polygons arranging along the tube axis also have two patterns: C_4_–C_8_ (blue) and C_6_–C_6_ (orange). Two C_4_–C_8_ patterns alternate with one C_6_–C_6_ pattern along the circumferential direction.

**Fig. 4:**
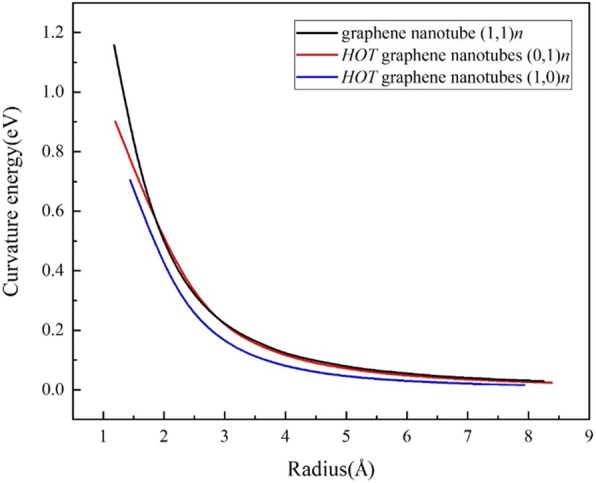
Curvature energies of the *HOT* graphene nanotubes and graphene nanotubes

To reveal the energy cost in rolling up a sheet into tubes, we define the curvature energy (Fig. 4) *E*_cur_ as:

$$ {E}_{\mathrm{cur}}={E}_{\mathrm{tube}}^{\mathrm{at}}-{E}_{\mathrm{sheet}}^{\mathrm{at}} $$

where ${E}_{\mathrm{tube}}^{\mathrm{at}}$ is the average energy of atoms in nanotube, and ${E}_{\mathrm{sheet}}^{\mathrm{at}}$ is the average energy of atoms in the 2D sheet. The (1,0) *n* tubes exhibit a lower energy cost than the graphene nanotubes while the (0,1) *n* tubes are nearly the same as the armchair graphene nanotubes except for several ultra-small tubes. Such results also suggest that it is possible to synthesize the *HOT* graphene nanotubes in experiments. It should be noted that tube (1,0)1 is too small because its diameter is even shorter than the bond length of carbon.

**Fig. 5:**
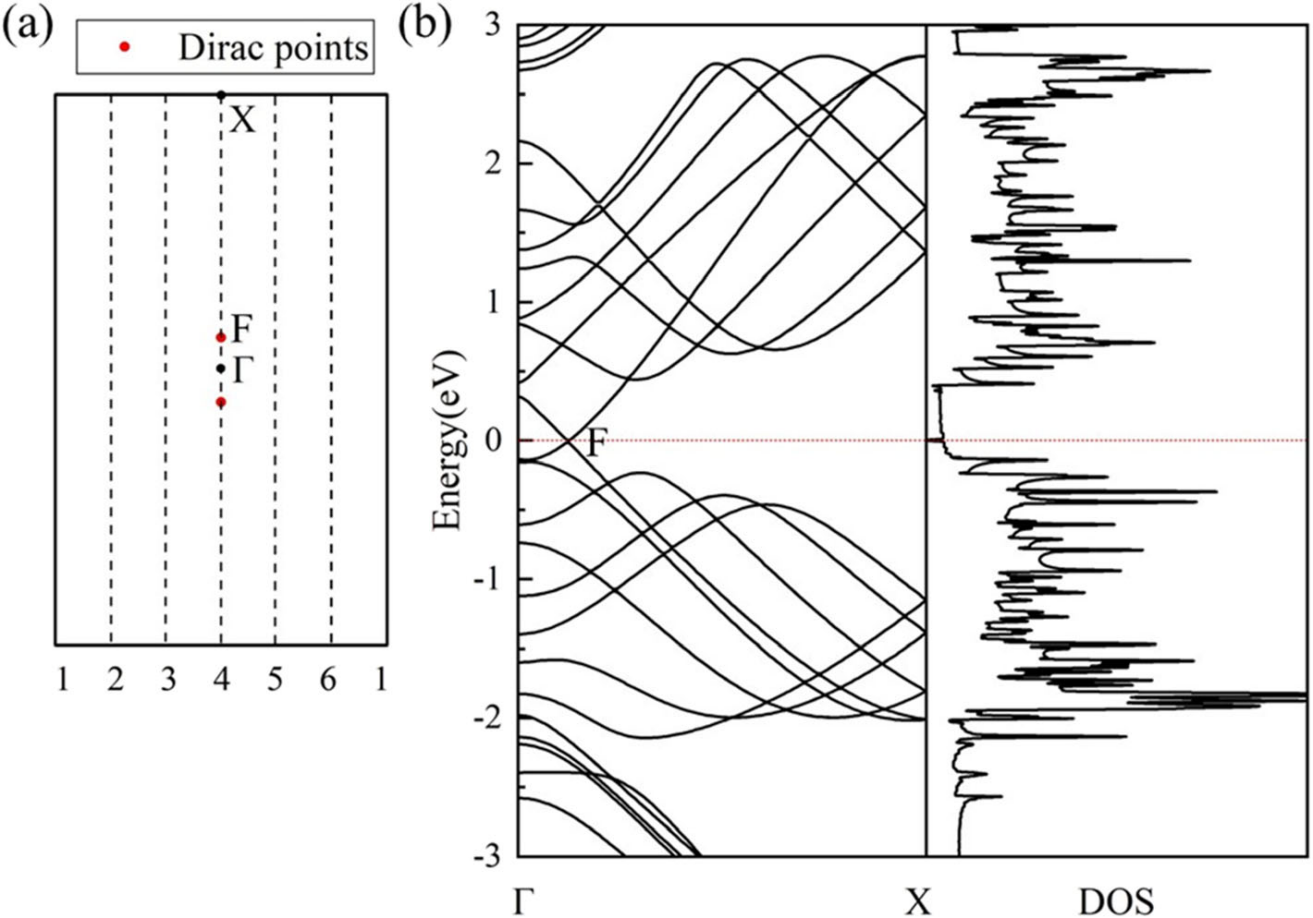
(**a**) The first BZ of the *HOT* graphene with allowed **k** lines (dash lines) for the (0,1)6 nanotube. F is the Dirac point. (**b**) The calculated band structures and DOS of (0,1)6 nanotube

The calculated electronic band structure and DOS of nanotube (0,1)6 (Fig. 5b) indicate a semimetallic character. When the *HOT* graphene sheet is rolled up into a nanotube, its 2D BZ reduces to 1D BZ as a result of the new periodic boundary conditions in nanotubes. The periodic boundary conditions along the circumferential direction of the tube only allow wave vectors “around” the nanotube circumference and these vectors are quantized [49]. The periodic boundary conditions along the nanotube axis remain the same as the 2D sheet, then the wave vectors remain continuous along the nanotube axis. According to the zone-folding scheme, the electronic band structure of a specific nanotube is given by the superposition of the electronic energy bands of the corresponding 2D sheet along the specifically allowed **k** lines [50]. As the quantized wave vectors in the middle of the BZ of the *HOT* graphene always cross the Dirac point (point F in Fig. 5a), a non-degenerate Dirac point (point F in Fig. 5b) and Dirac cone appears in the band structures of the (0,1) *n HOT* graphene nanotubes, resulting in the semimetallicity of all the (0,1) *n* nanotubes. Tube (0,1)6 is calculated to evaluate the semimetallicity of this set of (0,1) *n* nanotubes in Fig. 5. Band structure of the (0,1)6 *HOT* graphene nanotubes shows a crossing point of energy bands at the Fermi level and the corresponding DOS shows no states at the Fermi level which verifies the semimetallicity of the system. As the Dirac cone in (0,1) *n* tubes is originated from the *HOT* graphene sheet, the calculated coordinate of the Dirac point in the (0,1)6 nanotube is the same as that in *HOT* graphene sheet which is (0, 0.0585, 0). The calculated fermi velocity at the Dirac point in (0,1)6 nanotube is 6.76 × 10^5^ m/s, close to 6.27 × 10^5^ m/s in the *HOT* graphene sheet.

**Fig. 6:**
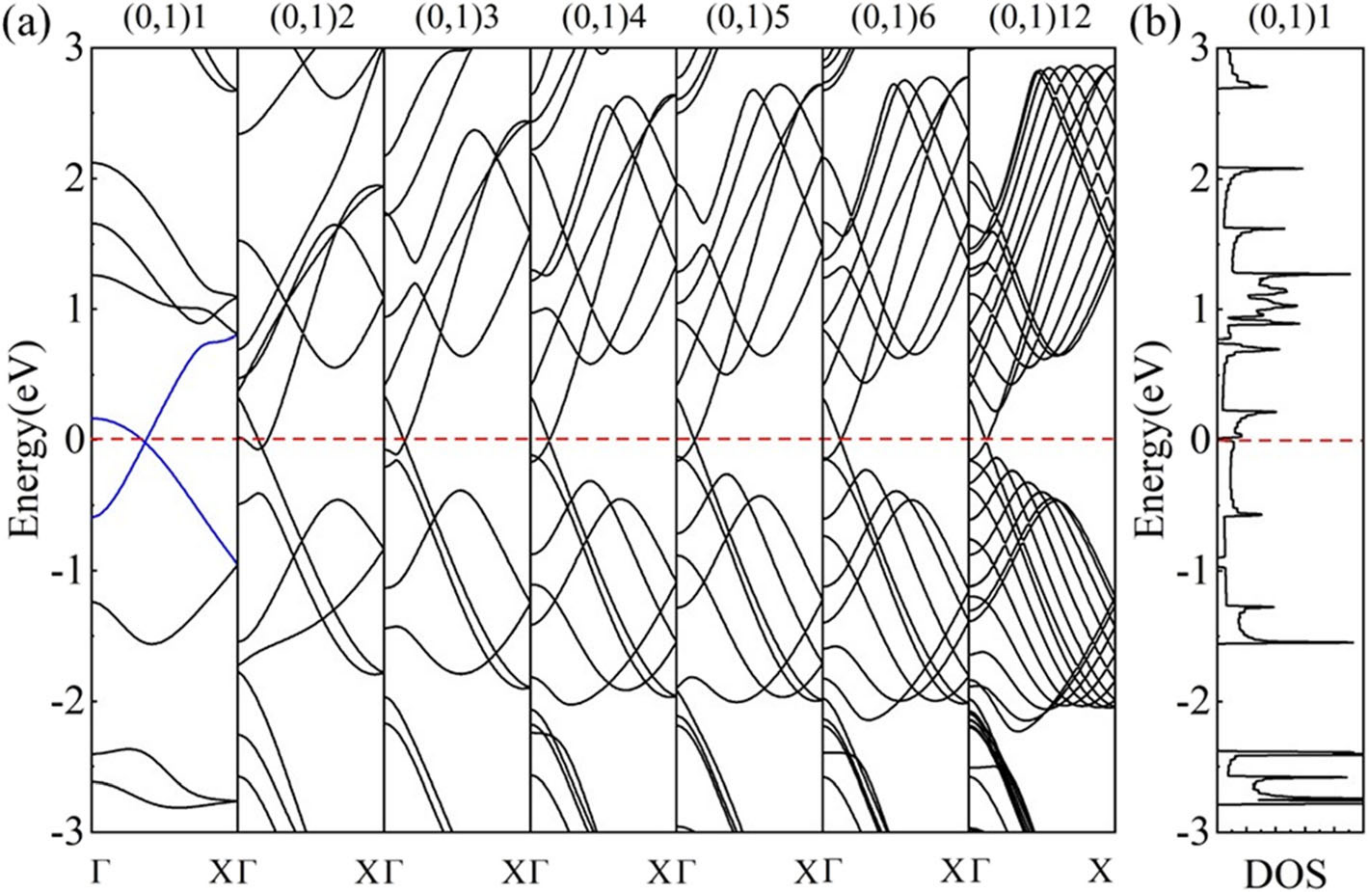
(**a**) Band evolution of (0,1) *n* nanotubes (*n* = 1, 2, 3, 4, 5, 6, 12); (**b**) DOS of the (0,1)1 nanotube

The band structure evolution of (0,1) *n* nanotubes with different tube radii (Fig. 6a) reveals that the *HOT* graphene nanotubes (0,1) *n* are semimetallic (*n* ≥ 3) and transform to metal (*n* = 2) and then turn back to semimetal (*n* = 1). Such a change under a small radius resulted from the so-called curvature effect [26]. In the situation of ultra-small nanotubes (e.g., (0,1)2 and (0,1)1), the curvature takes a non-negligible effect on the zone-folding scheme. The bond length and bond angle undergo a non-negligible change within the big curvature, which has an influence on the electronic band structure. The band structures of such nanotubes are no longer a simple superposition of energy bands on the allowed wave vectors. This change of bonds modifies the conditions that define the **k** point at which occupied and unoccupied bands do cross (at a point we label **k**_***F***_) and shift the **k**_***F***_ away from the original Dirac points, which is called the **k**_***F***_ shifting effect [24, 25]. As a result, in the (0,1) *n HOT* graphene nanotubes, the **k**_***F***_ shifts away from its original position (**k**_***HOT***_) in the *HOT* graphene sheet (point F in Fig. 5a). And the shifting direction of **k**_***F***_ is calculated to be along the allowed wave vector (dash lines in Fig. 5a), resulting in no change of the semimetallicity (Fig. 6a (*n* ≥ 3)). According to the calculated coordination of Dirac points, the **k**_***F***_ shifting effect becomes non-negligible at (0,1)5 with a tube radius of 5.988 Å, whose Dirac point (**k**_***F***_) shifts to (0,0.0626,0) from original point (**k**_***HOT***_) at (0,0.0594,0) in *HOT* graphene sheet. As the tube radius gets smaller, the **k**_***F***_ keeps shifting and reaches point (0,0.0712,0) in tube (0,1)3. In tube (0,1)2, the **k**_***F***_ shifts to (0,0.0835,0) where the Dirac point moves down below the Fermi level, resulting in a metallic system. The vanishing of the semimetallicity in (0,1)2 indicates a deviation from the **k**_***F***_ shifting effect in the (0,1) *n* nanotubes (*n* ≥ 3). Moreover, the (0,1)1 tube becomes semimetallic again in its band structure and the DOS (Fig. 6b). Our electron state analysis of the (0,1) *n HOT* nanotubes shows π states overlapping at *n* ≥ 2, which is usually considered the origin of the semimetallicity of graphene nanotubes [18, 24]. However, the corresponding electron state analysis of the *HOT* graphene nanotube (0,1)1 shows a σ-π hybridization that a low-lying σ* band intersects the Fermi level and joins the Dirac cone (blue lines in Fig. 6a). The coordination of Dirac point, which is (0,0.18345,0), also exhibits a distinction from other (0,1) *n HOT* graphene nanotubes. The calculated Fermi velocity is 4.47 × 10^5^ m/s, lower than 6.27 × 10^5^ m/s in *HOT* graphene sheet and values for other (0,1) *n* nanotubes (~6.76 × 10^5^ m/s). An obviously different shape of its band structure is also shown in Fig. 6a. All these characters verify that the semimetallicity of the *HOT* graphene nanotube (0,1)1 is originated from the σ-π hybridization. In summary, with the increasing curvature, the **k**_***F***_ shifting effect emerges at *n* = 5, becomes more effective at 4 ≥ *n* ≥ 2, and is finally replaced by the σ-π hybridization effect at *n* = 1.

**Fig. 7:**
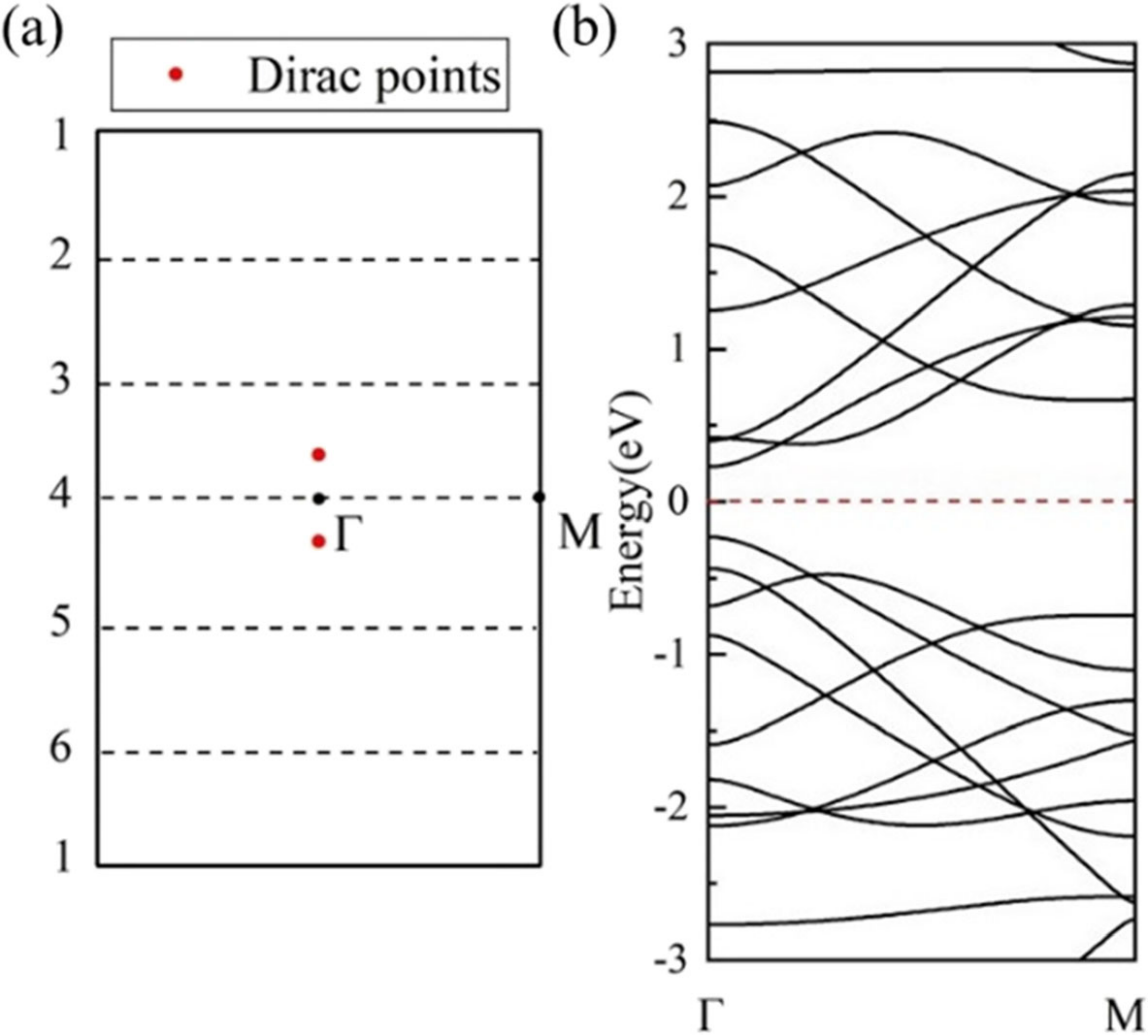
(**a**) The first BZ of the *HOT* graphene with allowed **k** lines (dash lines) for the (1,0)6 nanotube; (**b**) band structures of the (1,0)6 nanotube

**Fig. 8:**
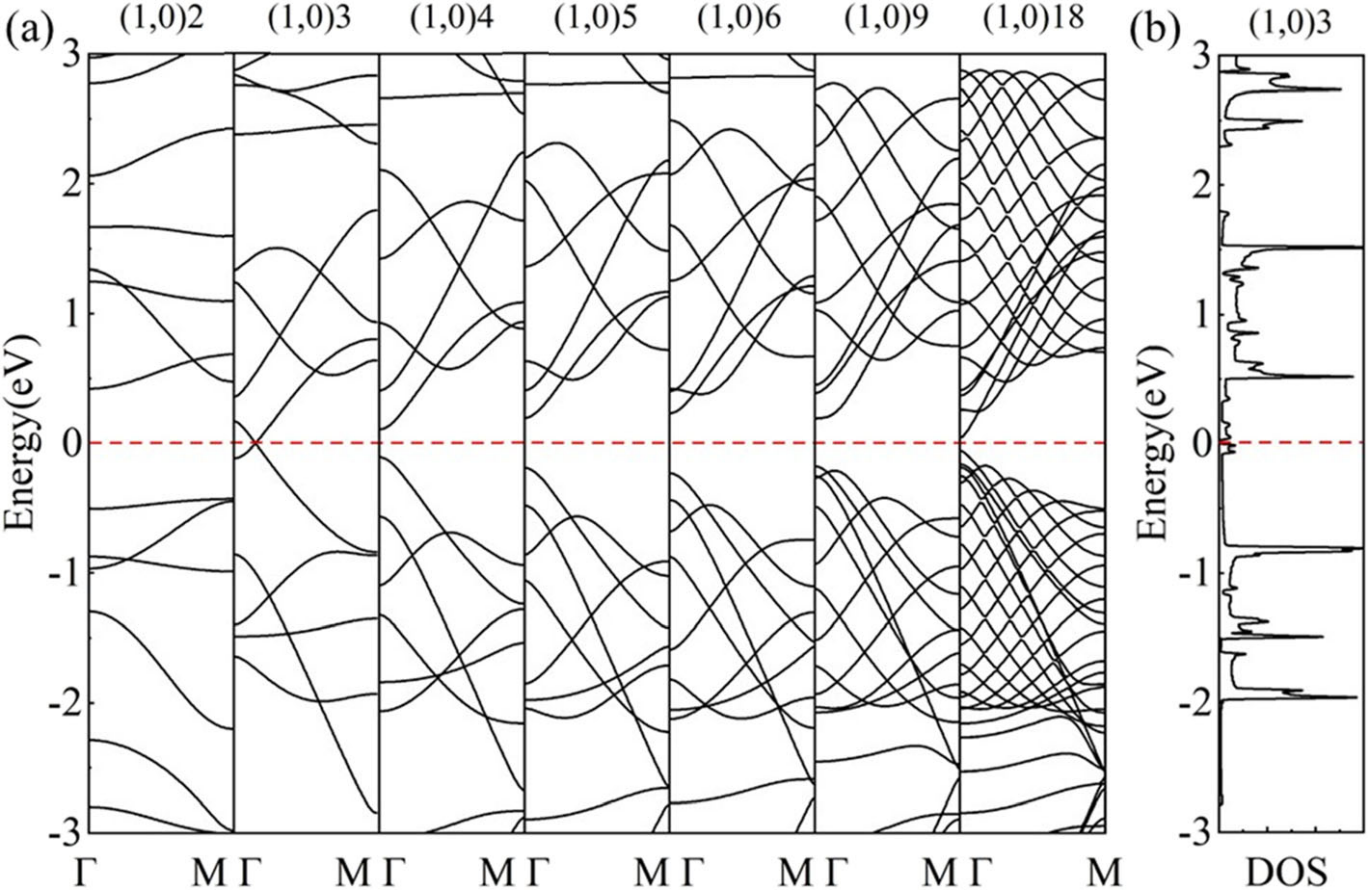
(**a**) Band evolution of *HOT* graphene nanotubes (1,0) *n* (*n =* 2, 3, 4, 5, 6, 9, 18); (**b**) DOS of the (1,0)3 nanotube

**Fig. 9:**
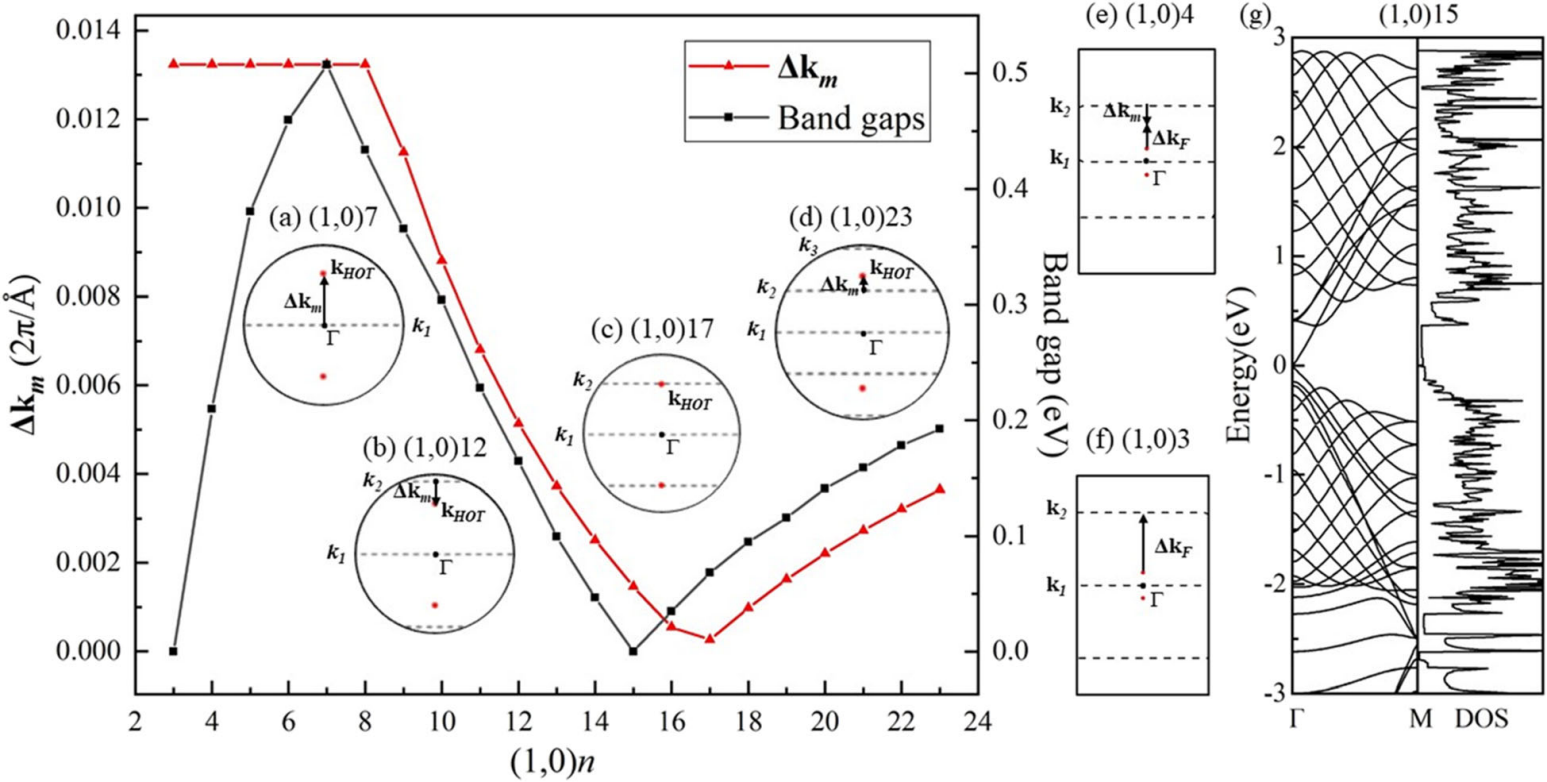
Calculated band gaps (black line) and distance **Δk**_***m***_ (red line) between **k**_***HOT***_ and the nearest **k** line in (1,0) *n* tubes. Inserted graphs are the BZ near the Γ point with allowed **k** lines **k**_***m***_ (*m* = 1, 2, 3) (dash lines) and the Fermi point of *HOT* graphene nanotubes **k**_***HOT***_ (red points) in tube (**a**) (1,0)7, (**b**) (1,0)12, (**c**) (1,0)17, (**d**) (1,0)23, (**e**) (1,0)4, and (**f**) (1,0)3. (**g**) Band structures and DOS of tube (1,0)15

In another rolling direction, the calculated band structure of (1,0)6 (Fig. 7b) shows semiconductivity. The 6 wave vectors (dash lines) are parallel to Γ-M, crossing the Г point in the middle of the BZ of *HOT* graphene (Fig. 7a), and open a 0.46 eV band gap as shown in the DOS (Fig. 7b). In this rolling direction, the allowed wave vectors in the middle of the BZ never include the Dirac points, which results in the nonzero band gaps in this set of nanotubes. The band evolution reveals a change of band gaps with different radii in Fig. 8a. The (1,0) *n HOT* graphene nanotubes are semiconductive (*n* ≥ 4). The valence band maximum (VBM) and conduction band minimum (CBM) get closer from tube (1,0)6 to tube (1,0)4 and then crosses with each other in tube (1,0)3 with a radius of 2.17 Å. This crossing point is exactly on the Fermi level located at (0.0791,0,0). The calculated DOS (Fig. 8b) presents 0 states at the Fermi level of (1,0)3, verifying the semimetallicity. When the tube radius decreases to (1,0)2, a 0.848 eV gap opens up again. The CBM and VBM of (1,0)2 are located at Γ point and M point, respectively, indicating an indirect band gap. This change in VBM implies a different origin for the semiconductivity in (1,0)2. A further study of the band-gap change in the (1,0) *n HOT* graphene nanotubes is shown in Fig. 9. The band gap evolution as a function of *n* (3 ≤ *n* ≤ 23) indicates that the band gap is adjustable with tube sizes. Also, instead of being monotonous, the dependence of the band gap as the tube size has a zigzag shape (Fig. 9 black curve). The global minimum at (1,0)15 presents a zero band gap. The semimetallicity of (1,0)15 is further confirmed by the band structure and DOS (Fig. 9g). From the zone-folding scheme, we know that the band structure of nanotubes is the superposition of the band structure of the 2D sheet along the corresponding quantized **k** lines [50]. Thus, the semimetallicity indicates that at least one of the allowed **k** lines (dash lines in Fig. 7a) intersects the Dirac points (red points in Fig. 7a) at *n* = 15. Otherwise, if the allowed **k** lines have a distance from the Dirac points (**k**_***HOT***_), a band gap will appear in nanotube. Furthermore, this distance between **k**_***F***_ and the **k** lines is proportional to the band gaps, as the band dispersion near the Dirac cone is linear [25]. The **Δk**_***m***_ measures the shortest distance between **k**_***F***_ and **k**_*m*_ lines of quantum number *m*. We calculated this distance **Δk**_***m***_ between the Dirac points (**k**_***HOT***_) and the allowed **k** lines in the *HOT* graphene and plot it (red line) together with the band gaps (black line) in Fig. 9. Firstly, all the allowed **k** lines have a quantum number *m*. When *n* ≤ 7 (e.g., Fig. 9a), the shortest distance (**Δk**_***m***_) is between the **k**_***HOT***_ and the first **k** line, **k**_**1**_, which sits at the Γ point constantly (insert graph (a) in Fig. 9). In this situation, **Δk**_***m***_ is constant as both **k**_**1**_ and **k**_***HOT***_ are constant independents of the tube size. However, as the allowed **k** lines become denser in larger nanotubes (7 ≤ *n* ≤ 17), the **k**_**2**_ becomes the nearest one to **k**_***HOT***_ (e.g. Fig. 9b). In this situation, the **k**_**2**_ approaches the Dirac point from the outer BZ with increasing tube radii; therefore, it shows a decline of **Δk**_***m***_ in Fig. 9 (7 ≤ *n* ≤ 17). *n* = 17 is a turning point where **k**_**2**_ almost intersects the Dirac point resulting in a local minimum of the distance **Δk**_***m***_ (Fig. 9c). As the radius keeps going up, the **k**_**2**_ traverse the **k**_***HOT***_ point and continues to move away from it to the Г point resulting in an increase of the distance again at 17 ≤ *n* ≤ 24 (e.g., Fig. 9d). At the same time, **k**_**3**_ is approaching the Dirac point. **k**_**3**_ gets closer to **k**_***HOT***_ than **k**_**2**_ and begins a new decrease in distance **Δk**_***m***_ at *n* ≥ 24. As the band gaps are proportional to this distance **Δk**_***m***_ [25], the band gap curve shows the same shape as the **Δk**_***m***_ plot (*n* ≥ 7). And it is revealed that the band gaps change in cycles: the **k**_***m***_ gets closer to the Dirac point (**k**_***HOT***_) causing a decline of band gap, then traverses the Dirac point resulting in a local minimum, then gets farther from the Dirac point causing a rising of band gap, and is finally replaced by the next line **k**_***m + 1***_ entering the next cycle. In summary, the reason why the band gaps are changing with tube size (*n* ≥ 7) is that the **k** lines are moving with different tube sizes, thereby the change the distance **Δk**_***m***_ between **k**_***HOT***_ and the allowed **k** lines which is proportional to the band gaps.

Though the **Δk**_***m***_ curve and the band gap curve have similarities in shape, the differences between them are also obvious, which is that the **Δk**_***m***_ plot shows a “delay” in change at *n* ≥ 7 and becomes completely different from the band gap plot at 3 ≤ *n* ≤ 7. The reason is that the Fermi point in *HOT* graphene nanotubes (**k**_***F***_) is assumed to have the same coordinate as the original Fermi point in *HOT* graphene (**k**_***HOT***_) in the preceding section where the band gap changing was explained. However, the Fermi point (**k**_***F***_) in nanotubes shifts away from the origin Fermi point (**k**_***HOT***_) in *HOT* graphene sheet under the curvature effect. Therefore, the **k**_***F***_ shifting (Δ**k**_***F***_ = **k**_***F***_–**k**_***HOT***_) effect contributes to the mismatches between the distance **Δk**_***m***_ and the band gaps. As calculated in the (0,1) *n* tubes (Fig. 6), the **k**_***F***_ in (1,0) *n* tubes also shifts to the outer BZ towards the symmetric point X under the curvature. Therefore, when the nearest **k**_***m***_ sits between the **k**_***HOT***_ and the Γ point, the distance **Δk**_***m***_ underestimates the band gap (e.g., 17 ≤ *n* ≤ 24 in Fig. 9d). Otherwise, the nearest **k**_***m***_ sitting at outer the **k**_***HOT***_ point, it results in an overestimate of the band gap (e.g., 7 ≤ *n* ≤ 17 in Fig. 9b). In small nanotubes (3 ≤ *n* ≤ 7), the **k**_***F***_ shifting effect is enhanced under the large growth rate of the curvature; consequently, it causes a drastic **k**_***F***_ shifting and changes the band gap. When the radius is getting smaller than *n* = 8, the **k**_***1***_ becomes the closest to the **k**_***HOT***_ indicating a constant **Δk**_***m***_. However, the **k**_***F***_ shifting effect is so strong to move the **k**_***F***_ farther from **k**_***1***_ but closer to the **k**_***2***_ (Fig. 9e). The **k**_***F***_ shifting effect wins the competition with the **k** lines moving and begins to determine the band gaps since then (*n* ≤ 7). The **k**_***F***_ keeps shifting to the **k**_***2***_ at a high velocity so that the distance between **k**_***F***_ and **k**_***2***_ gets smaller and smaller. Therefore, from (1,0)7 to (1,0)3, the band gap decreases (Fig. 9). At last, the **k**_***F***_ catches up with **k**_***2***_ and crosses it at *n* = 3(Fig. 9f). This crossing of a **k** line with the **k**_***F***_ results in the Dirac point in tube (1,0)3 that gives rise to the semimetallicity (Fig. 8) as discussed in the preceding sections. Further decrease of radius to *n* = 2 opens a 0.848 eV gap in the tube (1,0)2 (Fig. 8). This gap is so large and is considered out of the **k**_***F***_ shifting scheme, and hence is not plotted in Fig. 9. In summary, there is a competition mechanism between the **k** line moving and the **k**_***F***_ shifting in determining the band gaps. The **k**_***F***_ shifting effect leads in small tubes (7 ≥ *n* ≥ 3), while the **k** line moving leads in big tubes (*n* ≥ 8) where the **k**_***F***_ shifting effect is faded. The amount of the **k**_***F***_ shifting is estimated to be 0.0015 2π/Å at *n* = 15 and 0.0238 2π/Å at *n* = 3 as the band gaps are 0 eV where the shifted **k**_***F***_ point is on the allowed **k** lines. It can be seen that the **k**_***F***_ shifting is 15.86 times bigger in a small tube (tube (1,0)3) than in a big tube (tube (1,0)15).

**Fig. 10:**
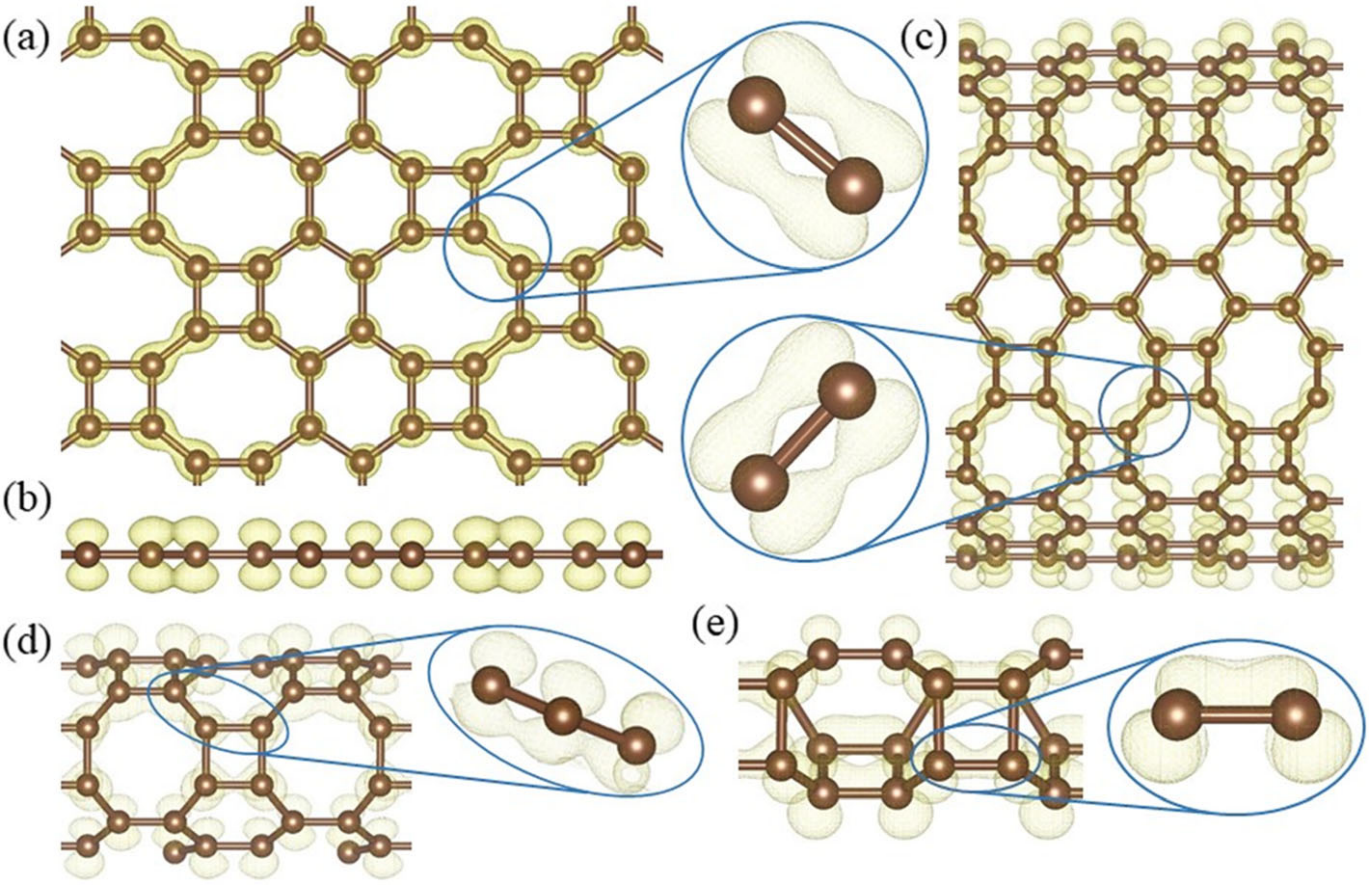
Band decomposed charge densities around the Fermi level of (**a**) *HOT* graphene, (**b**) the corresponding side view, and *HOT* graphene nanotubes (**c**) (0,1)6, (**d**) (0,1)2, and (**e**) (0,1)1 with corresponding enlarged side views

**Fig. 11:**
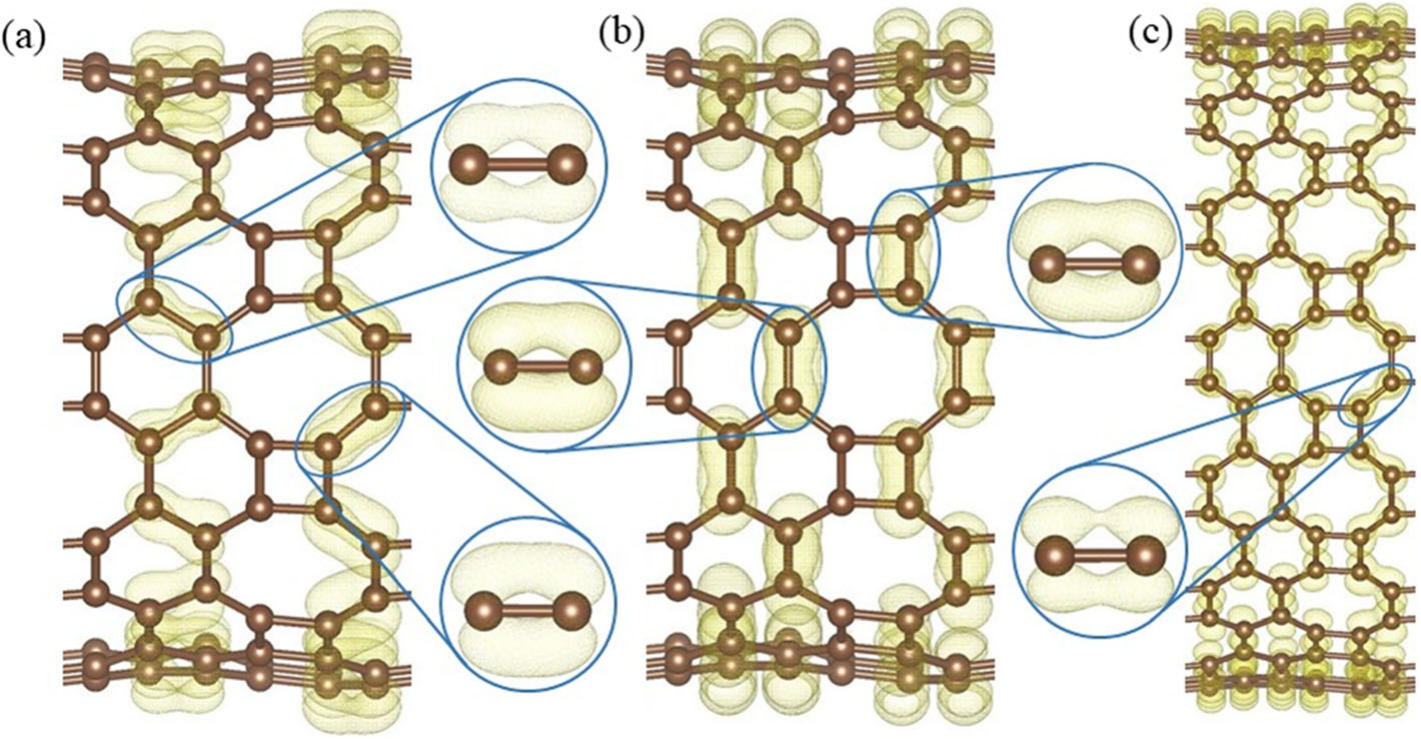
Band decomposed charge densities at (**a**) VBM, and (**b**) CBM of *HOT* graphene nanotube (1,0)9; (**c**) charge densities around the Fermi level of the *HOT* graphene nanotube (1,0)15 with the localized π bond in the enlarged side view

In *HOT* graphene, carbon atoms are all three-fold coordinated, thus the fourth valence electron plays a key role in its conductivity. The calculated band decomposed charge density ± 0.15 eV around the Fermi level (Fig. 10) shows the distribution of the electrons in the Dirac cone. Only the electrons on the 8–8 bonds (Fig. 10a) have an overlapping and the side view (Fig. 10b) shows that the electrons distribute perpendicularly to the *HOT* graphene sheet, which indicates that the Dirac cone consists of π states. Therefore, the electron overlapping on the 8–8 bonds (enlarged side view in Fig. 10a) is considered to be localized π states. In big nanotubes such as (0,1)6 (Fig. 10c), the charge density is similar to the *HOT* graphene sheet showing localized π bonds on 8–8 bonds (enlarged side view in Fig. 10c). As the radius decreases to (0,1)2, whose conductivity transforms to metal (Fig. 6), the 8–8 bonds show several deformations (Fig. 10d). Firstly, these states are no longer symmetric with respect to the tube wall. The overlapping of the π state outside the tube wall breaks apart while the π states inside keep overlapping with each other. Besides the 8–8 π bonds, new π bonds form on 4–8 in (0,1)2. These bonds are similar to the deformed 8–8 bonds: separated π states outside the tube wall and overlapping π states inside the tube wall. Every 4–8 bond connects two 8–8 bonds adjacent to it, forming a delocalized π overlapping inside the tube along the tube axis direction. The enlarged side view in Fig. 10d shows the connection between 4 and 8 and 8–8 bonds as a segment of the whole delocalized bond. Therefore, the metallicity in tube (0,1)2 can be attributed to the delocalized π overlapping in the 4–8 and 8–8 bonds along the tube axis direction which provides a pathway for the electrons to travel along the tube. When the radius keeps decreasing, the conductivity disappears and the (0,1)1 tube becomes a semimetal again (Fig. 6). Different from all the other (0,1) *n* tubes, the 8–8 overlapping (Fig. 10e) in nanotube (0,1)1 is totally broken up; instead, 4–8 overlapping and 4–6 overlapping plays the major role in the Dirac cones. These two bonds belong to two opposite edges in the same carbon tetragons and are arranged parallel to the tube axis. Furthermore, they are no longer π states. Based on the electronic state analysis, a σ-π hybridization takes place at the Fermi level under such a strong curvature in nanotube (0,1)1. It is verified by the charge density which shows the electron states distribute closely to the bond axis (enlarged side view in Fig. 10e). Strongly modified low-lying σ states are introduced at the Fermi level as discussed in the preceding sections (blue line in Fig. 6). Therefore, the σ-π hybrid states in the 4–8 and 4–6 bonds are considered the reason for the semimetallicity in (0,1)1, which is essentially different from the other semimetallic tubes (0,1) *n* (*n* ≥ 3). In another set of *HOT* graphene nanotubes, the band gaps show adjustability with different tube radius (Fig. 9). The charge densities also present an evolution with the tube radii in Fig. 11. The band decomposed charge density of tube (1,0)9 in Fig. 11a and b shows the localized π states overlapping in both the VBM and CBM. The VBM is contributed by π states on 6–6 bonds and 8–8 bonds (Fig. 11a). The CBM is contributed by π states on part of 4–8 and 6–8 bonds (Fig. 11b). The 4–6 bonds have no states on both of the VBM and CBM. When the (1,0) *n* tubes become semimetallic at some specific radii, such as *n* = 15, the VBM and CBM meet with each other. Band decomposed charge density ± 0.15 eV around the Fermi level of (1,0)15 nanotube show the different distribution of electrons from the semiconductive tubes. More importantly, it exhibits a similar distribution to the semimetallic *HOT* graphene sheet and (0,1) *n* tubes. The localized π bond of (1,0)15 only locates on the 8–8 bonds. This redistribution of electron in (1,0) *n* tubes causes the conductivity change.

## Conclusion

A new graphene allotrope named *HOT* graphene is constructed by carbon hexagons, octagons, and tetragons showing Dirac cone and high Fermi velocity, which implies that the honeycomb structure is not an indispensable condition for Dirac fermions to exist. The semiconductivity of *HOT* graphene is dependent on the localized π bonding. A corresponding series of nanotubes is rolled up from the *HOT* graphene sheet and shows distinctive electronic structures depending on the topology. The set of (0,1) *n* (*n* ≥ 3) *HOT* graphene nanotubes reveals a character of semimetallicity and Dirac cones that are composed by π states. A non-negligible **k**_***F***_ shifting along the allowed **k** line arises under the curvature effect when the tube radius gets smaller (3 ≤ *n* ≤ 5). However, the ultra-small nanotube (0,1)2 begins to deviate from the π state-based **k**_***F***_ shifting effect showing a transformation to metallicity. Finally, an σ-π hybridization takes the place of the π states at Fermi level in nanotube (0,1)1, where a low-lying σ* band intersection appears at the Fermi level and forms a semimetallicity again. Another set of tubes (1,0) *n* shows various band gaps (0~ 0.51 eV), which is continuously adjustable with the tube size. The band gaps of (1,0) *n* (*n* ≥ 3) nanotubes turn out to be determined by a competition mechanism between the **k** line moving and the **k*F*** shifting effect. The zone-folding approximation indicates a **k** line moving and results in the zigzag and periodical band gap changing curve in big tubes (*n* ≥ 8), while the **k*F*** shifting effect gets stronger and causes a dramatic decrease of band gaps in small tubes (7 ≥ *n* ≥ 3). Zero-gap semimetallic tubes appear periodically under the competition.

## Data Availability

Authors declare that the datasets used and/or analyzed during the current study are available to the readers and included in this article.

## Abbreviations

1D: One-dimensional
2D: Two-dimensional
3D: Three-dimensional
BZ: Brillouin zone
CBM: Conduction band minimum
DFT: Density functional theory
DOS: Density of states
GGA: Generalized-Gradient Approximation
*HOT*: Hexagons, octagons, and tetragons
PBE: Perdew–Burke–Ernzerh
VASP: Vienna Ab initio Simulation Package
VBM: Valence band maximum
